# Cancerous Protein Network That Inhibits the Tumor Suppressor Function of WW Domain-Containing Oxidoreductase (WWOX) by Aberrantly Expressed Molecules

**DOI:** 10.3389/fonc.2018.00350

**Published:** 2018-08-30

**Authors:** Chiemi Saigo, Yusuke Kito, Tamotsu Takeuchi

**Affiliations:** Department of Pathology and Translational Research, Gifu University Graduate School of Medicine, Gifu, Japan

**Keywords:** WW domain, WWOX, PPxY motif, cancer, aerobic glycolysis

## Abstract

Recent findings indicate that the WW domain-containing oxidoreductase (WWOX) is a tumor suppressor protein that contains two N-terminal WW domains and a central short-chain dehydrogenase/reductase domain. WWOX protein mediates multiple signaling networks that suppress carcinogenesis through binding of its first WW domain to various cancer-associated proteins, i.e., p73, AP-2γ, and others. Although the tumor suppressor property of WWOX is inarguable, WWOX is not inactivated in the manner characteristic of the canonical Knudson hypothesis. Impairment of both alleles of *WWOX* is thought to be a rare event, only occurring in a few cancer cell lines. How is the tumor suppressor function of WWOX impaired in cancer cells? Recent advances highlight that a small transmembrane protein possessing a PPxY motif, called TMEM207, and its relatives are aberrantly expressed in various cancer cells and hinder the tumor suppressor function of WWOX through inhibiting its WW domain. Here, we review the recent findings related to the pathobiological properties of TMEM207 and its relatives based on clinicopathological and experimental pathological studies.

## Introduction

WW domains are small protein modules with two conserved tryptophan (W) residues spaced at typically 35–40 amino acids in length (1). WW domain appeared to be slightly curved with an antiparallel β sheet to form a groove-like structure for ligand binding (2).

**Figure 1:**
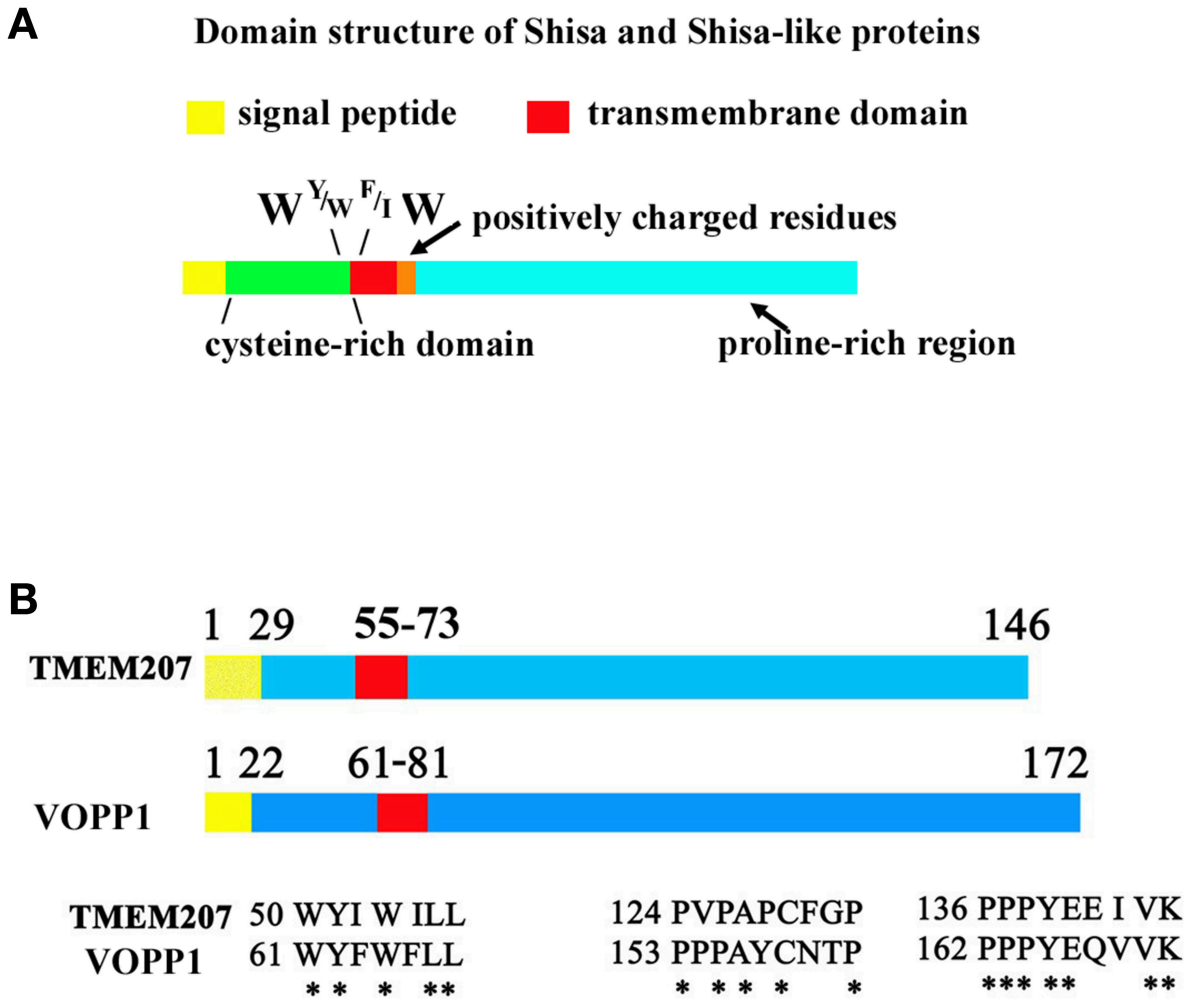
Protein sequence of Shisa/Shisa like family proteins and TMEM207. **(A)** Fundamental protein structure of Shisa/Shisa-like family proteins proposed by Pei and Grishin (9). In addition, we note the W, Y or W, F or I, W protein sequence at juxta-transmembrane domain. **(B)** Graphical depiction of homologous regions of TMEM207 and VOPP1. Note the PPxY motif, which binds WW-domain, at the C-terminus.

The WW-domain containing oxidoreductase (WWOX) is composed of two WW domains for signaling and a central short-chain dehydrogenase/reductase (SDR) domain for metabolism (3). The first N-terminal WW domain of WWOX binds protein ligands harboring motifs with core proline-rich sequences, not only PPXY (amino acid single-letter code; X is any amino acid) (PY) but also LPXY and LPXF motifs (where F is phenylalanine and L is leucine) (4–7). In contrast, the second WW domain contains two distinct amino acid residues within the WW binding pocket, compared to R25/W44 (R is Arginine/W is tryptophan) of the first N-terminal side WW domain, and exhibits no binding activity to PPXY ligands (8) (Figure 1A).

WWOX is believed to play a tumor suppressor role in carcinogenesis (10–13). WWOX interacts with various carcinogenesis-related molecules through its first N-terminal WW domain, probably interacting with growth regulatory proteins p73 (14), AP-2 γ (15), and others (16), thereby blocking cancer cell growth. Notably, WWOX suppresses p73 and AP-2γ oncogenic activity by sequestering them in the cytoplasm (14, 15). These findings indicate the important role of the WW domain in the tumor suppressor function of WWOX (Figure 1A).

WWOX is not inactivated in the manner characteristic of the classical Knudson hypothesis (17). *WWOX* is located on the common fragile site FRA16D, which has been linked to cancer-causing deletions and translocations (14, 18). In addition, loss of heterozygosity (15, 19) and promoter hypermethylation of *WWOX* (16, 17, 20, 21) may also be responsible for loss of the WWOX protein. However, impairment of both WWOX-encoding alleles is rare, occurring only in few cell lines (17). In mice, the susceptibility to N-nitrosomethylbenzylamine-induced forestomach tumorigenesis is higher in *Wwox*^+/−^ mice compared to that in wild type mice (22). However, many cancer cells escape loss of WWOX expression and express WWOX at various degrees, even robustly (23), as found in the data of The Human Protein Atlas (https://www.proteinatlas.org/ENSG00000186153-WWOX/pathology). WWOX is related to tumor progression rather than to initiation, and may not work as a highly penetrate tumor suppressor molecule (24).

The authors agree with the opinion that “despite many lines of evidence that suggest a role for loss of WWOX in the progression of cancer, our understanding of WWOX tumor-suppressive function is incomplete, as found in a recent remarkable report from Schrock et al. (25).

Recent advances suggest another molecular mechanism that could abrogate the tumor suppressor function of WWOX. A small transmembrane protein, TMEM207, appears to be aberrantly expressed and blocks the tumor suppressor function of WWOX through its PPxY motif (23, 26). TMEM207 competes with WWOX-interacting oncogenic molecules for binding to the WW domain of WWOX, thereby inhibiting the tumor suppressor function of WWOX. In addition, there are several cancer-promoting proteins with a molecular structure similar to that of TMEM207, such as VOPP1 (23, 27) (Figures 1B). There may be a tumor-associated family of transmembrane proteins, cancerous expression of which abrogates the tumor suppressor function of WWOX.

There are several outstanding reviews which highlight the protein networking, with which WWOX work as tumor suppressor molecule (28, 29).

In this review, we have summarized the recent findings of pathobiological protein networks that abolish the tumor suppressor function of WWOX in cancer cells, especially focusing on a small transmembrane protein, TMEM207.

## Mechanisms of WWOX inactivation in cancer cells

It is believed that the tumor suppressor function of WWOX is impaired by various molecular events in cancer cells. First, *WWOX* spans in the second most common human chromosomal fragile site, *FRA16D* at 16q23 (18). Chromosomal fragile sites are specific loci that preferentially exhibit gaps and breaks on metaphase chromosomes. Replication stress at *FRA16D* may participate in loss of WWOX protein expression. Second, the promoter region of *WWOX* can be hypermethylated, leading to gene silencing in several cancer cell types, i.e., *Helicobacter pylori* infection-related gastric cancer, intraductal papillary mucinous neoplasms of the pancreas (30, 31). Third, microRNAs (miRNAs), which can control gene expression, are also reported to be responsible for *WWOX* silencing. miR-134 expression contributes to head and neck carcinogenesis by targeting the WWOX (32). miR-153 regulates β-catenin activation through suppression of WWOX in hepatocellular carcinoma (33).

However, many cancer cells do express WWOX at various degrees, even robustly (23). Thus, our understanding of the tumor-suppressive function of WWOX remains inadequate (25).

Recent advances have shed light on the fourth molecular mechanism, which represses WWOX function through binding of undesirably expressed proteins to WWOX.

## TMEM207 and relatives as undesirable binding molecules to WWOX in cancer cells

In 2012, Pei and Grishin referred to several proteins, Shisa/Shisa-like, WBP1 (WW domain-binding protein 1), WBP1L (also known as OPAL1 (outcome predictor in acute leukemia 1), VOPP1 (vesicular over-expressed in cancer prosurvival protein 1), and more as STMC6 proteins (single-transmembrane proteins with conserved 6 cysteines) (9). STMC6 proteins contain a proline-rich C-terminal region. Although TMEM207 has no canonical cysteine-rich domain, TMEM207 is a single-transmembrane protein, which shares a ${\text{W}}^{\text{Y}}{\text{/}}_{\text{W}}^{\text{F}}{\text{/}}_{\text{I}}\text{W}$ amino acid motif in the extracellular portion adjacent to the cell membrane and C-terminal proline-rich domain, as found in STMC6 members (Figure 1). Percentage homology of representative STMC6 proteins and TMEM207 also suggests the close relation of these proteins (Supplementary Table 1).

TMEM207 and several STMC6 proteins bind to the WWOX protein. For example, WBP1, VOPP1, and TMEM207 bind to WWOX through its C-terminal PPxY motif (4, 23, 25, 27). Artificial mutation of PPxY motif abolishes the binding of WBP1 or TMEM207 to WWOX. VOPP1, also known as ECop (EGFR-coamplified and overexpressed protein), is overexpressed in esophageal squamous cell and gastric adenocarcinoma.

Recently, Bunai et al. reported that overexpressed TMEM207 co-localized and bound to WWOX in oral squamous cell carcinoma, especially glycogen-rich cancer cells, by using an *in situ* proximal ligation assay (26). They also demonstrated that TMEM207 promoted aerobic glycolysis of oral squamous cell carcinoma by abrogating the WWOX-mediating regulation of HIF1α protein. Since the *in situ* proximity ligation assay is well accepted to detect sub-cellular spatial molecular protein-protein interactions (34), this finding again verified the interaction of TMEM207 and WWOX in cancer cells.

VOPP1 promotes cell proliferation and migration and thus might serve as a putative oncogene. Very recently, Lallemand et al. (27) reported that VOPP1 physically interacts with WWOX. Upon binding, WWOX is recruited to the VOPP1-containing lysosomal compartment. This recruitment inhibits WWOX-mediated apoptosis at least in part by preventing WWOX-p73 interaction (27). Although further studies are required, TMEM207 and other members of the STMC6 family may constitute a novel transmembrane protein family that hinder the WWOX tumor suppressor function.

## Physiological cellular and tissue expression and biological properties of TMEM207

It was in 2003 that “The secreted protein discovery initiative” first identified TMEM207 as a novel transmembrane protein (35). Later, TMEM207 appeared to be also localized in the endoplasmic reticulum by bioinformatic analysis (36). TMEM207 expression is strictly regulated in human tissues and cells. *TMEM207* mRNA is found in human kidney (36) and brain microvascular endothelial cells (37); low levels are found in other cells and tissues, except intestinal goblet cells (23, 38).

Maeda et al. demonstrated the binding and co-localization of intelectin-1 and TMEM207 in cytoplasm by an *in situ* proximal ligation assay (38). Notably, siRNA-mediated down-regulation of TMEM207 increases polyubiquitination and proteasome degradation of intelectin-1, subsequently decreasing intelectin-1 secretion (38). These data indicate that TMEM207 may participate in the quality and quantity control of intelectin-1 at the endoplasmic reticulum.

**Figure 2:**
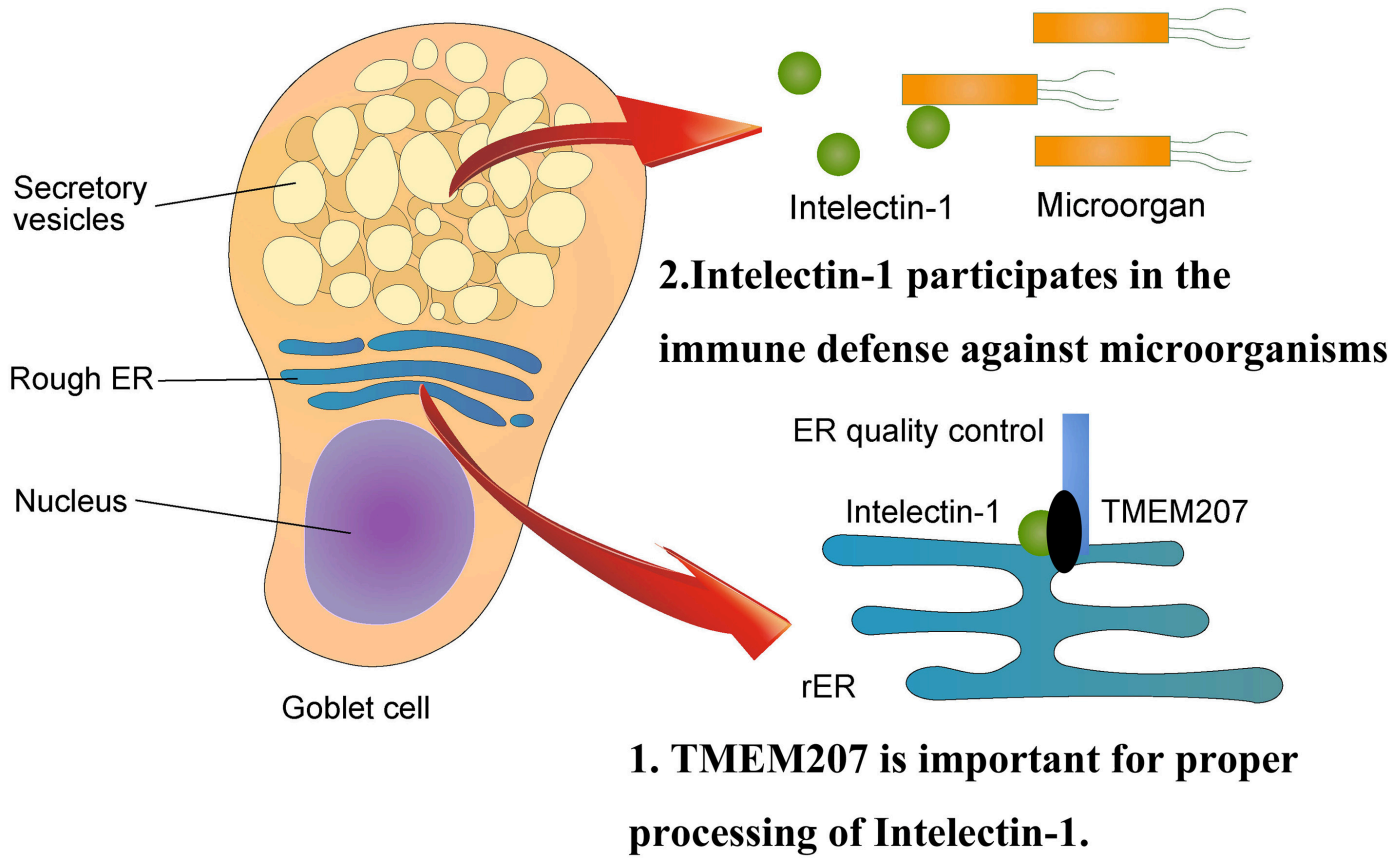
Physiological aspects of TMEM207. At the endoplasmic reticulum, TMEM207 participates in the quality control of intelectin-1, which recognizes glycan epitopes found exclusively on microbes and plays a role in intestinal innate immunity through assisting phagocytic clearance of microorganisms. TMEM207 may participate in intestinal innate immunity by promoting the proper secretion of intelectin-1. TMEM207 and its relatives harbor a canonical PPxY motif to bind the WW domain of WWOX.

Human intelectin-1 recognizes multiple glycan epitopes found exclusively on microbes and plays a role in intestinal pathogen-host interaction through assisting phagocytic clearance of microorganisms (39, 40). TMEM207 may participate in intestinal innate immunity through appropriate secretion of intelectin-1 (Figure 2). Since intelectin-1 is also called omentin, which appears to be an adipokine with insulin-sensitizing properties (41), TMEM207 may also play metabolic roles through proper processing and secretion of intelectin-1 (42).

## Pathological aspects related to aberrant TMEM207 expression in mice

### Relation to cutaneous adnexal tumors

Kito et al. generated several transgenic mice lines in which murine TMEM207 was highly expressed under a truncated (by ~200 bp) proximal promoter of the murine intestinal trefoil factor (ITF) gene (also known as Tff3) (43). One of these TMEM207-overexpressing transgenic mouse lines spontaneously exhibited a cutaneous adnexal tumor as found in human Brooke-Spiegler syndrome (a genetic condition associated with predisposition to cutaneous adnexal tumors) (44). In this line, the ITF-TMEM207 construct was inserted into a major satellite repeat sequence in chromosome 2, in which no definite coding molecule was found. In addition, cutaneous adnexal tumors were found, although less frequently, in three other transgenic mouse lines. Notably, hair follicle bulge cells exhibit overexpression of TMEM207 in this transgenic mouse line. It is well established that hair follicle bulge cells are multipotent stem cells that support hair follicle cycling and repopulation (45). Taken together, the data suggest that the origin of cutaneous adnexal tumors, which has long been uncertain, may be a transformed hair follicle bulge cell. Further study exploring whether loss of WWOX participates in cutaneous adnexal tumorigenesis is needed.

### Relation to renal cystogenesis

Kito et al. also reported renal cystogenesis in a TMEM207-Tg mouse line, in which the transgene (ITF-TMEM207) was inserted into a basic helix-loop-helix leucine zipper lesion of the microphthalmia-associated transcription factor (*Mitf*) gene, thereby disrupting the expression of MITF proteins (46). MITF protein regulates key transcriptions for survival and differentiation of pigmented cells (47). Mutational studies in mice have shown that *Mitf* is essential for melanocyte and eye development (48) However, renal cystogenesis has not been reported in mice with MITF mutations. In addition, the 13 other strains harboring the same transgene (ITF-TMEM207) do not exhibit renal cystogenesis. A combination of loss of MITF and overexpression of TMEM207 may be important to renal cystogenesis in this novel transgenic mouse line.

WWOX is also strongly expressed in in the distal convoluted tubules and proximal collector ducts (49). Ludes-Meyers et al. reported that WWOX-knockout mice suffered from severe metabolic defects and suggested a role of WWOX in acid/base balance (50). However, the exact physiological role of WWOX in the kidney remains unclear. It should be determined whether loss of WWOX function leads to kidney cystogenesis.

According to “The Human Protein Atlas,” human kidney tissue expresses several WW domain-containing proteins, i.e., BCL2 associated athanogene 3 (BAG3), WW Domain Containing Transcription Regulator 1 (WWTR1), and IQ motif containing GTPase activating protein 2 (IQGAP2). Further studies, which aim to examine the relation between TMEM207 and these WW domain-containing proteins expressed in the kidney, are needed to unravel the physiological property of TMEM207 in the kidney.

### Relation to gastrointestinal carcinogenesis

Until now, no reports describing TMEM207 transgenic mice that develop gastrointestinal cancer were available. We speculate that overexpression of TMEM207 may not be enough to cause gastrointestinal carcinogenesis, but it might promote gastrointestinal cancer progression by enhancing the cancer cell invasion activity, as described in Gastric and Colonic Carcinogenesis section.

## Pathological aspects related to aberrant TMEM207 expression in humans

### Gastric and colonic carcinogenesis

In 2004, Aqeilan RI et al. reported that loss of WWOX protein expression was found in 65% of primary gastric adenocarcinoma specimens. They also reported that loss of heterozygosity at the WWOX locus was found in 31% of gastric adenocarcinoma specimens. Interestingly, Helicobacter pylori infection, a major risk factor for gastric cancer, increased hypermethylation of WWOX (29). These findings indicate that impaired expression of WWOX leads to gastric carcinogenesis.

In 2012, we identified TMEM207 as selectively expressed in collagen gel-invading gastric signet-ring cell carcinoma cells (SRCCs) (23). A subsequent study unraveled that TMEM207 promotes the invasion of gastric SRCCs in a manner dependent on its C-terminal PPxP motif. Interestingly, TMEM207 bound to WWOX and impair the WWOX-mediated repression of Matrigel invasion activity of gastric SRCCs.

As described in section TMEM207 and Relatives as Undesirable Binding Molecules to WWOX in Cancer Cells, TMEM207 is expressed in intestinal goblet cells for the precise quality control of intelectin-1. TMEM207 is not expressed in intact gastric epithelium, whereas TMEM207 is strongly expressed in intestinal metaplastic gastric epithelium with well-formed goblet cells. Gastric intestinal metaplasia is well characterized as an intermediate precancerous gastric lesion in the gastric cancer cascade of *Helicobacter pylori H*.-associated chronic active gastritis, atrophic gastritis, intestinal metaplasia, dysplasia, and adenocarcinoma. During this gastric cancer cascade, gastric epithelium may gain TMEM207 expression.

Maeda et al. also found that TMEM207 is strongly expressed in mucinous colon cancer, which harbors abundant mucin-rich cytoplasm, similar to gastric SRCCs and the intestinal metaplastic epithelium (38). We propose that highly expressed TMEM207 may competitively bind to the WW domain of WWOX, thus inhibiting the tumor suppressor function of WWOX during carcinogenesis in digestive tract cancers.

### Oral squamous cell carcinogenesis with relation to aerobic glycolysis

Another example of an oncogenic property of highly expressed TMEM207 is found in invasive oral squamous cell carcinoma (OSCC). Very recently, Bunai et al. found that TMEM207 was highly expressed in 40 of 90 OSCC samples but not in neighboring non-tumorous epithelial tissues (26). Overexpression of TMEM207 is significantly associated with nodal metastasis and poor prognosis of OSCC patients. Notably, co-localization of TMEM207 and WWOX in invasive OSCC cells, especially glycogen-rich ones, was demonstrated by an *in situ* proximal ligation assay. *In situ* proximal ligation assay, originally developed by Fredriksson and colleagues in 2002, is now well accepted to be a dependable technique to see the spatial cellular protein-protein binding. Combined together with the finding of co-immunoprecipitation assay, which showed the binding of TMEM207 to WWOX as its PPxY motif dependent manner, pathobiological link of TMEM207 and WWOX may be occurred in various cancer cells.

Bunai et al. further demonstrated that TMEM207 contributes to tumor progression in OSCC, possibly via promoting aerobic glycolysis (26). Currently, WWOX is believed to modulate cancer metabolism (51). WWOX is downregulated under hypoxic conditions, while WWOX decreases HIF1α protein levels without affecting transcription of HIF1α under normal oxygen conditions. WWOX directly binds to HIF1α in a WW domain-dependent manner and increases HIF1α hydroxylation, which is known to lead to the degradation of HIF1α protein under normal oxygen conditions. It is well characterized that cancer cells preferably undergo aerobic glycolysis, the ‘Warburg effect’. In other words, glycolysis is markedly upregulated in cancer cells even in the presence of oxygen. HIF1α plays a critical role in aerobic glycolysis through activating its downstream factor Glut1. Therefore, impaired WWOX function may participate in the “Warburg effect” through HIF1α stabilization, which increases the expression of Glut1 and other aerobic glycolytic metabolism-related molecules in cancer invasion microenvironments.

Overexpression of TMEM207 may participate in cancer metabolism to promote cancer growth.

## Prospects of regulating TMEM207 expression in cancer cells

Notably, when examining TMEM207 in cBioPortal for Cancer Genomics (http://www.cbioportal.org/), it was revealed that TMEM207 is amplified in most cancers, especially in lung squamous cell carcinoma, esophageal, ovary, and head and neck cancers. Moreover, Kaplan Meier plotter analysis, http://kmplot.com/analysis/, indicated that high expression of TMEM207 correlates with worse prognosis in gastric cancer.

The recent findings described above indicate that highly expressed TMEM207 could be a therapeutic target for patients with TMEM207-expressing cancers. Since TMEM207 is also expressed in the cell surface membrane of dysplastic and cancer cells, we are now developing antibody-based approaches targeting several cancers. Furthermore, the PPxY motif of TMEM207 using small molecules, which targets the neighboring lesion of the PPxY motif, might be promising.

## Concluding comments

Recent advances demonstrate that several cancer cell types gain ectopic overexpression of TMEM207 during carcinogenesis, and then abolish the tumor suppressor function of WWOX through competitively binding with its WW domain. We propose that this inhibitory mechanism of TMEM207 to WWOX might lead to carcinogenesis if cancer cells continue to express the WWOX protein. Three important points have yet to be revealed. First, detailed molecular mechanisms that occur upon binding of TMEM207 to WWOX should be investigated. What cancer-related pathway molecule(s) would be affected by this phenomenon? Is it the p73 signaling pathway? Next, TMEM207 expression should be rigorously examined in most cancers or malignant tumors, including rare malignant tumors, i.e., types of sarcoma. Last, the exact molecular mechanism that is responsible for aberrant TMEM207 expression remains unclear. To our knowledge, the promoter or enhancer regions of *TMEM207* also remain undetermined.

## Author contributions

All authors listed have made a substantial, direct and intellectual contribution to the work, and approved it for publication.

### Conflict of interest statement

The authors declare that the research was conducted in the absence of any commercial or financial relationships that could be construed as a potential conflict of interest.

## Supplementary material

Supplementary Table 1 % identity of deduced amino acid sequence of human Shisa/Shisa-like family proteins, VOPP1, WBP1, Shisa-like-1, TMEM92, and TMEM207 determined by SIM program, https://web.expasy.org/sim/ with comparison matrix: BLOSUM30, Number of alignments computed: 30, Gap open penalty: 25, and Gap extension penalty: 0.
