# Supplementary material for: Cancerous Protein Network That Inhibits the Tumor Suppressor Function of WW Domain-Containing Oxidoreductase (WWOX) by Aberrantly Expressed Molecules
Source: Front Oncol. 2018 Aug 30;8:350. doi: 10.3389/fonc.2018.00350 (PMC6125347; doi:10.3389/fonc.2018.00350)
Supplement: Supplementary Table 1 — % identity of deduced amino acid sequence of human Shisa/Shisa-like family proteins, VOPP1, WBP1, Shisa-like-1, TMEM92, and TMEM207 determined by SIM program, https://web.expasy.org/sim/ with comparison matrix: BLOSUM30, Number of alignments computed: 30, Gap open penalty: 25, and Gap extension penalty: 0. [file Table_1.DOCX]

|  | Shisa-like-1 | TMEM207 | VOPP1 | WBP1 | TMEM92 |
| --- | --- | --- | --- | --- | --- |
| Shisa-like-1 |  | 14.8% | 15.7% | 11.6% | 11.7% |
| TMEM207 |  |  | 16.5% | 12.1% | 17.0% |
| VOPP1 |  |  |  | 17.1% | 21.1% |
| WBP1 |  |  |  |  | 19.5% |
| TMEM92 |  |  |  |  |  |
